# Glycated Soy β-Conglycinin Nanoparticle for Efficient Nanocarrier of Curcumin: Formation Mechanism, Thermal Stability, and Storage Stability

**DOI:** 10.3390/foods11223703

**Published:** 2022-11-18

**Authors:** Zijun Wang, Jingjing Xu, Fuyun Ji, Huihui Liu, Chuyan Wang, Shuizhong Luo, Zhi Zheng

**Affiliations:** 1 Key Laboratory for Agricultural Products, Processing of Anhui Province, School of Food and Biological Engineering, Hefei University of Technology, Hefei 230601, China; 2 School of Biology, Food and Environment, Hefei University, Hefei 230601, China

**Keywords:** soy β-conglycinin, dextran, curcumin, glycation, thermal stability

## Abstract

In this study, soy β-conglycinin (7S) was glycated with dextran of different molecular masses (40, 70, 150, 500 kDa) by the dry-heating method to synthesize soy β-conglycinin-dextran (7S-DEX) conjugates. The curcumin (Cur) loaded nanocomplexes were prepared based on 7S-DEX conjugates by a pH-driven self-assemble strategy to enhance the solubility and thermal stability of curcumin. Results showed that the 7S-150 conjugates (glycated from 7S with dextran (150 kDa)) could remain stable in the pH 3.0–pH 8.0 range and during the heat treatment. The results of fluorescence quenching and FT-IR indicated that glycated 7S were combined with curcumin mainly by hydrogen bonding and hydrophobic interaction, and 7S-150 conjugates had higher binding affinity than natural 7S for curcumin. The loading capacity (μg/mg) and encapsulation efficiency (EE%) of 7S-150-Cur were 16.06 μg/mg and 87.51%, respectively, significantly higher than that of 7S-Cur (12.41 μg/mg, 51.15%). The XRD spectrum showed that curcumin was exhibited in an amorphous state within the 7S-150-Cur nanocomplexes. After heating at 65 °C for 30 min, the curcumin retention of the 7S-150-Cur nanocomplexes was about 1.4 times higher than that of free curcumin. The particle size of 7S-150-Cur nanocomplexes was stable (in the range of 10–100 nm) during the long storage time (21 days).

## 1. Introduction

Curcumin (Cur), as a natural lipophilic polyphenol extracted from the rhizomes of turmeric (*Curcuma longa*), exhibited a broad range of medicinal activities such as anti-oxidant, anti-inflammatory, and antiviral properties [1]. Nevertheless, the poor water solubility and thermal stability of curcumin extremely limit its wide application in the food industry [2]. So far, many methods have been proposed to improve the water-dispersibility and stability of curcumin such as encapsulation in emulsions [3], inorganic nanoparticles [4], and proteins [5]. Among these, the use of biopolymer nanoparticles made from proteins to construct a closed environment to protect curcumin has generated a great interest in the field of function foods.

Protein-based nanoparticles have been commonly used as nanocarriers for curcumin because of their excellent biosafety, biocompatibility, and biodegradability [6]. Many proteins, for example, whey proteins, soy protein, and bovine serum albumin simply bind bioactives to the surface through hydrophobic interactions, resulting in a limited curcumin loading amount, and accelerated the instability of nanocomplexes [7,8,9]. Some proteins, for example, β-casein [10] and soy β-conglycinin [8], have a unique nanostructure that can be disassembled to expose more binding sites for curcumin, and then reassembled to encapsulate curcumin within the self-assembled structure, which enabled them to be developed as high-encapsulation-performance nanocarriers for curcumin by the self-assembly technology.

Soy β-conglycinin (7S), as one of the major storage globulins of soy protein, is a trimer comprised of three subunits: α′ (~71 kDa), α (~67 kDa), and β (~50 kDa). The subunits of 7S are connected by non-covalent interactions [11]. Based on this unique characteristic, 7S can be disassembled and reassembled induced by many methods such as the pH-driven strategy [7], ethanol-assisted strategy [12], and urea-assisted strategy [8]. Meanwhile, 7S has been successfully used to prepare nanocomplexes with curcumin and have exhibited a high encapsulation efficiency [7,8]. However, as a protein, native 7S is highly vulnerable to environmental stresses (including extreme pH and high temperature), which has limited the application of natural 7S as a nanocarrier for hydrophobic nutrients in the food industry [13].

Recently, a large amount of reliable evidence has shown that glycation is an effective, convenient, and secure strategy for improving protein function [13,14]. Maillard conjugates have some unique characteristics compared with the natural proteins including eminent solubility, thermal stability, and pH stability [5]. Polysaccharides bound to the surface of proteins could provide intensive steric repulsion to inhibit the aggregation of nanoparticles at pH values near the protein pI or upon heating treatment. In the past study, Xu et al. [13] have reported that glycation could effectively increase the thermal stability of 7S nanoparticles. Meanwhile, our recent study has demonstrated that 7S-DEX conjugates have a similar pH-driven self-assemble ability as natural 7S [7]. Results showed that encapsulation within 7S-DEX conjugates could greatly improve the sustained-release property and antioxidant activity of curcumin. However, the lack of evaluation of heat and storage stability limited the application of 7S-DEX-Cur nanocomplexes for oral administration and the delivery of bioactive compounds.

Therefore, the objective of this work was to investigate the efficacy and stability of glycated 7S as a nanocarrier for curcumin. 7S was glycated with different molecular masses of dextran (40, 70, 150, 500 kDa) at the same dry-heating condition as the prepared 7S-DEX conjugates. The obtained 7S-DEX conjugates were characterized with SDS-PAGE, degree of glycation, heat stability, and pH stability. The 7S-150 conjugates exhibited a desirable pH stability and thermal stability was used to prepare nanocomplexes with curcumin. Then, the formation mechanism and physicochemical properties of the developed 7S-150-Cur nanocomplexes were characterized through intrinsic fluorescence spectrum, FT-IR, XRD, and TEM. Finally, the thermal and storage stability of 7S-150-Cur nanocomplexes were also investigated.

## 2. Materials and Methods

### 2.1. Materials

Soy β-conglycinin (7S) was separated from defatted soy flour (Shandong Baiyao Industrial and Commercial Co. Ltd., Shandong, China) based on the method reported by Nagano et al. [15]. Curcumin (purity > 98%), dextran (40, 70, 150, 500 kDa), and ophthaldialdehyde (OPA) were obtained by Sinopharm Chemical Reagent Co. Ltd. (Shanghai, China). All other chemicals used were of analytical grade.

### 2.2. Preparation of 7S-DEX Conjugates

Based on the process of Xu et al. [13], 7S and dextran (DEX) of different molecular masses (40, 70, 150, 500 kDa) were dispersed in deionized water at a concentration of 20 mg/mL, respectively. After that, 7S and dextran solutions were intermixed in a 1:1 mass ratio. The obtained aqueous mixtures were freeze-dried at −50 °C for 48 h. The lyophilized samples were heated to 60 °C in a sealed desiccator that contained a saturated KBr solution for 3 days. The resulting 7S-dextran conjugates were denoted as 7S-40, 7S-70, 7S-150, and 7S-500, respectively.

### 2.3. Preparation of 7S-Cur and 7S-150-Cur Composite Nanoparticles

According to the report of Wang et al. [6], 7S and 7S-150 conjugates were dispersed in distilled water at a 5 mg/mL protein concentration. After complete hydration, the solutions were made up to pH 12.0 and equilibrated at 25 °C for 1 h. Then, curcumin crystals were mixed with 7S or 7S-150 conjugate solution, and the concentration of curcumin was 0.1 mg/mL. After stirring for a further 30 min at 600 rpm, the samples were acidified to pH 7.0 and centrifuged at 6500× *g* for 20 min. The prepared 7S-Cur and 7S-150-Cur nanocomplexes were stored in a 4 °C refrigerator or freeze-dried.

### 2.4. Characterization of 7S-DEX Conjugates

#### 2.4.1. SDS-PAGE Analysis

A precast gel (Bio-Rad, 4–20% polyacrylamide, 12 wells) was used to perform SDS-PAGE analysis [16]. Each well was loaded with 10 μL of 7S or 7S-DEX conjugates in an aqueous solution (2 mg/mL protein concentration). After running, Colloidal Coomassie G-250 was used to dye the gel, and the gel was scanned with an Imager Scanner III (EU-88, Epson, Japan).

#### 2.4.2. Measurement of Degree of Glycosylation (DG%)

The OPA method was used to determine the DG% of samples [13]. Briefly, 4 mL of the OPA reagent was mixed with two hundred microliters of 7S or 7S-DEX conjugate solution, and then incubated under 25 °C for 2 min. Then, the absorbance of the mixture was measured at 340 nm by UV–Vis spectrometer (UV2600, Shimadzu, Japan). The DG% was assessed according to the formula below:

(1) $$DG\left(\%\right)=\left[\frac{\left(A_{0}-A_{S}\right)}{A_{0}}\right]\times 100$$

where A_0_ and A_s_ refer to the absorbance of the untreated 7S and glycated 7S, respectively.

#### 2.4.3. Evaluation of 7S-DEX Conjugates Stability

The 7S and 7S-DEX conjugate suspension was adjusted to different pH values (3–8), and then stirred at 25 °C for 30 min. The particle size of the 7S and 7S-DEX conjugates was measured by Malvern Zetasizer 2000 (Malvern Instruments, Southborough, MA, USA) to evaluate the pH stability [17]. Furthermore, the 7S and 7S-DEX conjugate solutions was heated at 90 °C for 30 min, and the particle size of the nanoparticles was measured to assess the thermal stability of the 7S and 7S-DEX conjugates [17].

### 2.5. Characterization of 7S-Cur and 7S-150-Cur Nanocomplexes

#### 2.5.1. Fluorescence Quenching Measurement

According to the process of Wu et al. [18], the interaction between 7S/7S-150 and curcumin was explored by fluorescence spectra. The intrinsic fluorescence spectra of 7S/7S-150 (0.1 mg/mL protein concentration) with curcumin (10, 20, 40, 50, 60, 70, and 40 μmol/L) were studied at 25 °C by a Shimadzu spectrophotometer (RF-6000, Japan). The fluorescence intensity was measured in an emission wavelength range of 300–450 nm and the excitation wavelength was 280 nm. The width of the excitation and emission slits were both fixed at 5 nm.

#### 2.5.2. Z-Average Diameter, Zeta-Potential, and Polydispersity Index

The Malvern Zetasizer 2000 was applied to determine the Z-average diameter, zeta-potential, and polydispersity index of the 7S-Cur and 7S-150-Cur nanocomplexes [19]. The samples were diluted to 1 mg/mL with PBS (10 mM, pH 7.0), and then measured at room temperature.

#### 2.5.3. Determination of Loading Amount (LA) and Encapsulation Efficiency (EE%)

The LA and EE% of the 7S-Cur and 7S-150-Cur nanocomplexes were measured as follows [20]. The 0.2 mL of the fresh nanocomplex solution was fully mixed with 3 mL of ethanol to withdraw the encapsulated curcumin, and then centrifugation at 10,000× *g* for 15 min. The absorbance of the supernatant was assayed by a UV754N UV–Vis spectrophotometer (Precision & Scientific Instrument, Shanghai, China) at 420 nm as Y and used to convert into the curcumin concentration by the standard curve of curcumin–ethanol solutions: Y = 0.1556X − 0.0171 (Y is the absorbance of samples; X is curcumin concentration, mg/mL; R^2^ = 0.999). The ratio of encapsulated curcumin to total protein is defined as LA (μg per 100 mg), and the EE% was determined based on the proportion of the amount of encapsulated curcumin to the total amount.

#### 2.5.4. Fourier Transform Infrared Spectroscopy (FT-IR)

The FTIR spectroscopy of free curcumin, 7S, 7S-150, 7S-Cur, and 7S-150-Cur were measured by a Spectrum 100 Fourier transform spectrophotometer (PerkinElmer, Inc., Waltham, MA, USA) [9]. KBr was mixed with each powdered sample in the proportion of 1:100. The mixtures were milled to fine powder and pressed into slices. The spectra were acquired by scanning in the wavenumber range of 500 to 4000 cm^−1^ at a resolution of 4 cm^−1^.

#### 2.5.5. X-ray Diffraction (XRD)

The XRD spectra of free curcumin, 7S, 7S-150, 7S-Cur, and 7S-150-Cur were measured by an XRD-7000S diffractometer (Shimadzu, Tokyo, Japan). The diffraction curves of the samples were acquired by scanning the angle range of 5° to 60° (2θ) at a speed of 5°/min [21].

#### 2.5.6. Transmission Electron Microscopy (TEM)

The nanocomplexes were diluted to a 20 μg/ mL concentration with deionized water, and then dripped on 230-mesh copper grids. After drying naturally, the samples were imagined by TEM (JEM-1230, JEOL, Tokyo, Japan) at 80 kV to assess the microstructure of the 7S-Cur and 7S-150-Cur nanocomplexes [22].

#### 2.5.7. Thermal Stability

Thermal stability of free or encapsulated curcumin was represented by measuring the curcumin retention ratio after heating treatment [23]. Briefly, free curcumin and freshly prepared 7S-Cur/7S-150-Cur suspensions were heated at high temperatures (75, 85, 95 °C) for 30 min, and then immediately cooled to 25 °C. After that, the residual curcumin in the solutions was measured as aforementioned (Section 2.5.3), and recorded to represent the thermal stability of curcumin.

#### 2.5.8. Storage Stability

Freshly prepared nanocomplexes were stored at room temperature for 21 days. The particle size change of the nanocomplexes was monitored every 7 d to evaluate the storage stability of 7S-Cur and 7S-150-Cur [24]. The particle size of the nanoparticles was measured according to Section 2.5.3.

### 2.6. Statistical Analysis

All experiments were repeated three times. The resulting data were analyzed by analysis of variance (ANOVA) and Duncan’s test using IBM-SPSS software version 25. The results are represented as the mean ± standard deviation, and *p* < 0.05 was considered as statically meaningful.

## 3. Results and Discussion

### 3.1. S-DEX Conjugate Characterization

#### 3.1.1. DG% Analysis

The OPA method was applied to measure the degree of grafting (DG%) value of the 7S-DEX conjugates [25]. As shown in Table 1, with the molecular weight of dextran increasing from 40 to 500 kDa, the DG% of the glycated proteins was gradually decreased from 16.19% to 6.79%, indicating that the dextran molecular weight significantly affected the binding efficiency between 7S and dextran. This result could be explained by the higher molecular weight dextran having a stronger steric hindrance that limited the accessibility of the reducing sugar end. Thus, the increasing dextran molecular weights would lead to a monotonic decrease in the conjugation efficiency [26]. In the study of ovalbumin-dextran conjugates, similar results could also be found [26].

#### 3.1.2. SDS-PAGE Analysis

**Figure 1:**
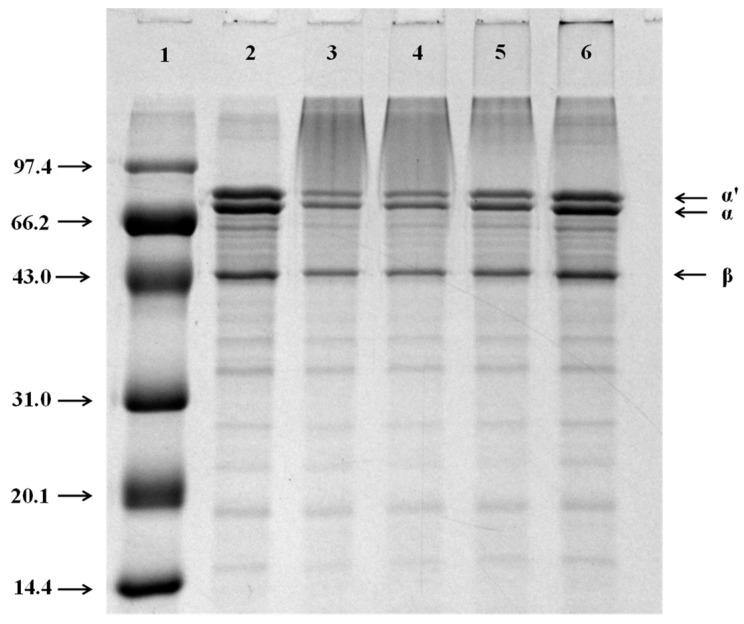
SDS-PAGE profiles of 7S and 7S-DEX conjugates obtained at 60 °C and RH 79%. Lanes: 1, Maker; 2, native 7S; 3, 7S-40; 4, 7S-70; 5, 7S-150; 6, 7S-500. 7S: soy β-conglycinin; DEX: dextran; 40: dextran (40 kDa); 70: dextran (70 kDa); 150: dextran (150 kDa); 500: dextran (500 kDa).

The successful conjugation of 7S with dextran was also verified by the results of SDS-PAGE (Figure 1.). As anticipated, native 7S was composed of three subunits: α’ (~71 kDa), α (~67 kDa), and β (~50 kDa), which was affirmed in Lane 2. Compared with native 7S (Lane 2), a new band occurred at the top of Lanes 3–6 (bands of 7S-DEX conjugates), which clearly demonstrated that 7S and dextran were successfully connected by covalent bonds, and the resulting 7S-DEX conjugates had a larger molecular weight. Furthermore, with the increasing molecular weight of dextran (Lanes 3–6), the high-molecular-weight new band at the top zone gradually became paler, while the characteristic bands (approximately 67, 71, 50 kDa) of 7S were correspondingly much denser. This result suggests that the increase in the dextran molecular weight (in the range of 40 to 500 kDa) led to a decrease in the production of 7S-DEX conjugates. The reasons might be attributed to the lower accessibility of the reducing end of dextran, which has a high molecular weight, resulting in the conjugation efficiency of 7S and dextran decreasing, which were consistent with the results of DG%.

#### 3.1.3. pH Stability of 7S-DEX Conjugates

**Figure 2:**
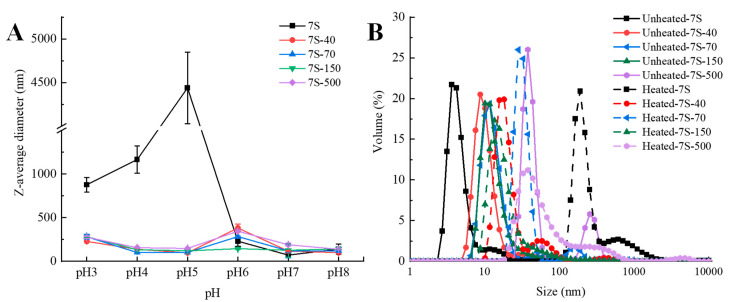
The influence of pH on the Z-average diameter of the 7S and 7S-DEX conjugates (7S-40, 7S-70, 7S-150, 7S-500). (**A**), and the changes in the particle size of the 7S and 7S-DEX conjugates (7S-40, 7S-70, 7S-150, 7S-500) before and after heating at 90 °C for 30 min. (**B**), 7S: soy β-conglycinin; DEX: dextran; 40: dextran (40 kDa); 70: dextran (70 kDa); 150: dextran (150 kDa); 500: dextran (500 kDa).

Many food products are susceptible to pH changes and thermal treatments during processing, so it is vital to inspect the influence of pH and high temperature on the stability of nanocomplexes [6]. Particle size variation in the 7S/7S-DEX conjugates with and without extreme treatment were measured to characterize the pH stability of the nanoparticles. As illustrated in Figure 2A, the Z-average diameter of native 7S was greatly affected by different pH treatments, and sharply increased at pH 5.0, which could be attributed to the decreased electrostatic repulsion close to the isoelectric point of 7S (pI = 4.8) [11]. However, the particle size of the 7S-DEX conjugates changed slightly in the pH 3.0–pH 7.0 range, indicating that the 7S-DEX conjugates have an eminent stability to aggregation against pH (especially in the vicinity of pI), which was due to the hairy polysaccharide protrusions on the 7S-150 conjugates, provided strong steric hindrance that inhibited the aggregation between nanoparticles [17,27]. Liu et al. [17] indicated the attachment of dextran was caused strong steric hindrance, which inhibited the aggregation of the whey protein isolate under unfavorable conditions. Among all of the obtained 7S-DEX conjugates, the Z-average diameter of the 7S-150 nanoparticles was the most stable in the pH range examined, suggesting that the 7S-150 conjugates had an eminent pH stability. Compared with 7S-40 and 7S-70, 7S-150 had larger dextran chain lengths, which could provide greater steric hindrance to stabilize the nanoparticles at extreme pH. However, due to the long dextran chain lengths, 7S-500 exhibited a limited DG% (only 6.79%), so most proteins in the obtained 7S-500 nanoparticles might not have had dextran attached to their surfaces, resulting in 7S-500 being more likely to cluster than 7S-150.

#### 3.1.4. Thermal Stability of 7S-DEX Conjugates

The particle size distribution of the 7S and 7S-DEX conjugates were determined to explore the effects of glycation on the nanoparticle stability against heat treatment. In Figure 2B, most of the particles in the native 7S solution were smaller than 15 nm before the heat treatment. Due to glycation with dextran, the particle size of the 7S-DEX conjugates ranged from 15 to 100 nm, which is apparently higher than that of 7S. However, after the heat treatment, the particle sizes of glycated 7S (<100 nm) were significantly smaller than that of native 7S (100–1000 nm), which evidently confirms that glycation with dextran could inhibit the thermal aggregation of 7S. Furthermore, the heating treatment almost had no influence on the particle size distribution of the 7S-150 conjugates, indicating that the 7S-150 nanoparticles have an excellent thermal stability (similar to the reports by Xu et al. [13]). Therefore, in the following sections, 7S-150 conjugates were selected to prepare the nanocomposite with curcumin.

### 3.2. Quenching of 7S and 7S-150 Fluorescence by Curcumin

The alterations in protein intrinsic fluorescence may indicate changes in their surrounding microenvironment, which were induced by ligand binding or protein conformational transition. Therefore, the intrinsic fluorescence spectrum has been extensively utilized to elucidate the binding mechanism of polyphenols and proteins [18]. Figure 2 showed the fluorescence spectra (excitation at 280 nm, 25 °C) of 7S/7S-150 in different concentrations of curcumin. It could be observed that the incorporation of curcumin led to a concentration-dependent quenching of 7S/7S-150 intrinsic fluorescence intensity, suggesting that curcumin could bond with proteins through hydrophobic interactions to form 7S-Cur/7S-150-Cur nanocomplexes, resulting in fluorescence quenching [11]. Li et al. [28] have also reported similar results.

Fluorescence quenching could be divided into dynamic quenching and static quenching, the difference being the dependence on temperature [29]. Dynamic quenching is the spread of the quencher onto the fluorophore during the lifetime of the excited state, and static quenching represents the formation of fluorophore-quencher complexes. Whether dynamic or static quenching, the mechanism can be elucidated by using the Stern–Volmer equation:

(2) $$\frac{F}{F_{0}}=1+K_{SV}\left[Q\right]=1+K_{q}\tau_{0}\left[Q\right]$$

Herein, F and F_0_ are the fluorescence intensities of 7S with and without curcumin, respectively; [Q] is the curcumin concentration; Ksv is the Stern–Volmer quenching constant; Kq is the bimolecular quenching constant; τ_0_ (10^−8^ s) is the average fluorescence lifetime of the biopolymer without quencher [30]. The magnitude of the quenching rate constant (Kq) is usually used to estimate the quenching mechanism, and the maximum value of the collisional quenching constant is 2 × 10^10^ M^−1^s^−1^.

**Figure 3:**
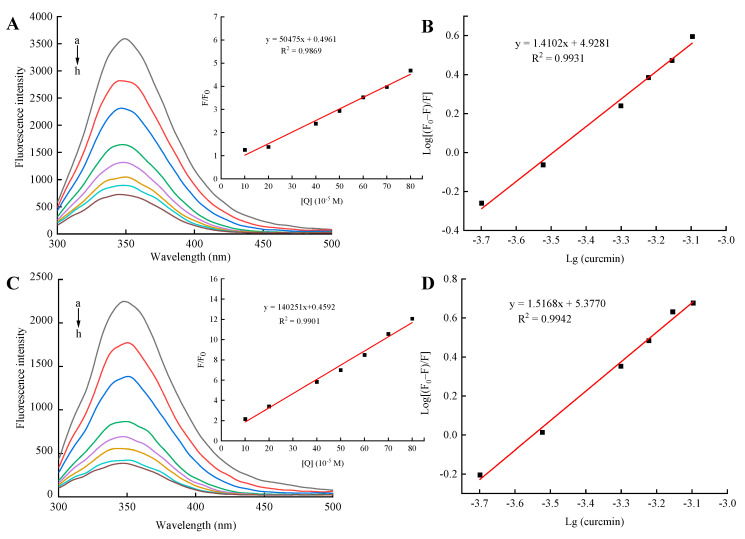
Fluorescence emission spectra of 7S (**A**) and 7S-150 (**C**) in the presence of various concentrations of curcumin. The inset corresponds to Stern-Volmer plots for the quenching of 7S/7S-150 by curcumin. Conditions: Temperature, 25 °C; λex, 280 nm; 7S, 5 mg/mL; curcumin, 0-80 μM (a–h). Logarithmic plots for the quenching of 7S (**B**) and 7S-150 (**D**) fluorescence by curcumin. 7S: soy β-conglycinin; 150: dextran (150 kDa).

As illustrated in Figure 3A,B, the Stern–Volmer plots of 7S and 7S-150 quenched by different concentrations of curcumin all exhibited a favorable linear relationship. The Kq values for 7S and 7S-150 were 0.51 × 10^13^ and 1.40× 10^13^ M^−1^·s^−1^, respectively, which were all 2–3 orders of magnitude larger than the collisional quenching constant (2 × 10^10^ M^−1^s^−1^), suggesting that the addition of curcumin induced a static fluorescence quenching. Therefore, 7S-Cur/7S-150-Cur nanocomplexes were formed by the complexation of the ground state molecules of the fluorophore in protein with curcumin [28].

The number of binding sites and binding constant of 7S-Cur/7S-150-Cur nanocomplexes in the static quenching reaction can be calculated by the following equation:

(3) $$\log\left[\frac{F_{0}-F}{F}\right]=\mathrm{logK}+\mathrm{nlog}\left[Q\right]$$

where F_0_, F, and [Q] are given in Equation (3); K is the binding constant of 7S/7S-150 and curcumin; *n* represents the number of binding sites. Figure 3B,D exhibits the plots of log ($\frac{F_{0}-F}{F}$) vs. log[Q] for 7S and 7S-150, respectively. It can be seen that each plot showed good linearity (R^2^ > 0.99). The *n* values of 7S-Cur and 7S-150-Cur were 1.41 and 1.51, respectively, indicating that the bonding sites of curcumin and dextran on 7S were different, so glycation had no influence on the number of curcumin binding sites (≈1.5) per molecule of 7S or 7S-150. Furthermore, the binding constant of 7S-150 to curcumin was 2.38 × 10^5^ M^−1^, much higher than that of 7S (0.85 × 10^5^ M^−1^). The increased binding affinity could be attributed to glycation with dextran, which resulted in the polarization of 7S molecules being promoted, and the hydrogen bonding and van der Waals forces between 7S and curcumin were strengthened [9]. Fu et al. found that glycation with glucose could enhance the binding affinity of bovine serum albumin to curcumin [9].

### 3.3. Characterization of 7S-Cur and 7S-150-Cur Nanocomplexes

#### 3.3.1. Z-Average Diameter, Polydispersity Index (PDI), and ζ-Potential of 7S-Cur and 7S-150-Cur Nanocomplexes

The results in Table 2 show the ζ-potential, Z-average diameter, and polydispersity index (PDI) of the nanocomplexes. The Z-average diameter of 7S-150-Cur was 84.37 nm, significantly higher than that of 7S-Cur (75.11 nm) due to the attachment of dextran in the 7S-150 nanoparticles. The value of PDI <0.3 suggests that the particles exhibited a relatively ideal dispersity [31]. As illustrated in Table 2, the PDI value of 7S-Cur and 7S-150-Cur were 0.24 and 0.27, respectively, lower than 0.30, indicating that the nanocomplexes all exhibited a homogeneous size distribution. Usually, composite nanoparticles with uniform or homogeneous dispersion demand a high absolute value of the ζ-potential to preserve the intense electrostatic repulsion among nanoparticles, whereas the absolute value of the ζ-potential of 7S-150-Cur (7.88 mV) was significantly lower than that of 7S-Cur (17.77 mV). This result could be due to the 7S-150-Cur nanocomplexes being coated by nonionic dextran. The distance between the aqueous and the charged groups of protein was increased due to the attachment of dextran, resulting in electrostatic interactions between the nanoparticles and the surrounding water being weakened [32]. The uniform size distribution of 7S-150-Cur could be due to the strong steric hindrance supplied by dextran, which inhibited the agglomeration of nanocomplexes. These findings are consistent with the description of Yi et al. [33].

#### 3.3.2. Encapsulation Efficiency (EE%) and Loading Amount (LA) of 7S-Cur and 7S-DEX-Cur Nanocomplexes

EE% and LA of the active ingredients is an important factor in evaluating the functional properties of nutrient nanocarriers. The EE% and LA of the 7S-Cur nanocomplexes were 87.51% and 16.06 μg/mg (Table 2), respectively, which was significantly lower than that of the 7S-150-Cur nanocomplexes (51.15%, 12.41 μg/mg). This is in agreement with the results of fluorescence quenching in Section 3.2, suggesting that glycation with dextran could increase the bonding affinity of 7S to curcumin. In past studies, Chen et al. [34] and Fu et al. [9] also found that glycation could improve the EE% and LA and of proteins to curcumin.

#### 3.3.3. FTIR Analysis

**Figure 4:**
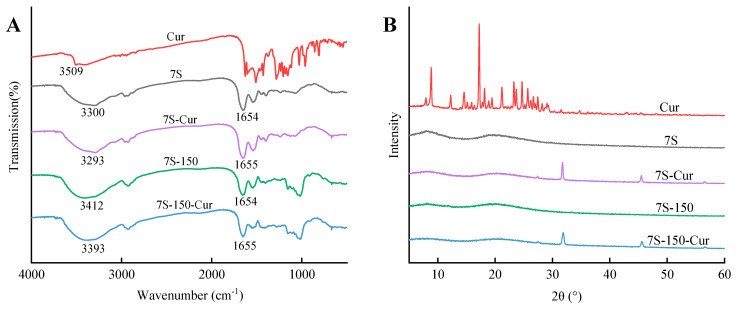
Fourier transform infrared (FTIR) spectra (**A**) and X-ray diffraction (XRD) patterns (**B**) of free curcumin, 7S, 7S-150, 7S-Cur, and 7S-150-Cur nanocomplexes. 7S: soy β-conglycinin; 150: dextran (150 kDa); Cur: curcumin.

The driving force of the formation of the 7S-Cur and 7S-150-Cur nanocomplexes was analyzed by FTIR. As shown in Figure 4A, free curcumin exhibited characteristic absorbance peaks at 3509 cm^−1^ (O–H stretching), 1628 (conjugated ketone), 1370 cm^−1^ (–CH bending vibration), and 1283 cm^−1^ (C-O stretching) [21]. In the spectra of the 7S and 7S-150 conjugates, the bands at 3300~3340 cm^−1^, 1700~1600 cm^−1^, and 1600~1500 cm^−1^ corresponded to O–H stretching, the amide I band, and amide II band, respectively. The characteristic absorbance peaks in the 750–1500 cm^−1^ region found in the free curcumin spectrum disappeared in the spectra of the 7S-Cur and 7S-150-Cur nanocomplexes, suggesting that curcumin was successfully embedded in the complex nanoparticles. In addition, after complexation with curcumin, the peak of amide I in the 7S/7S-150 spectra shifted from 1654 cm^−1^ to 1655 cm^−1^. Meanwhile, the peaks of O–H in the spectra of 7S (3300 cm^−1^) and 7S-150 (3412 cm^−1^) shifted to 3293 cm^−1^ and 3393 cm^−1^ in the spectra of their curcumin loaded nanocomplexes, respectively. These results suggest that the 7S/7S-150 conjugates were combined with curcumin mainly by hydrogen bonding and hydrophobic interaction to form the 7S-Cur and 7S-150-Cur nanocomplexes.

#### 3.3.4. XRD Analysis

The XRD spectrum was applied to explore the influence of encapsulation within the 7S or 7S-150 conjugates on the crystallographic diffraction of the curcumin phase. Typically, amorphous curcumin can be released more effectively than crystalline ones [35]. As shown in Figure 4B, the 7S and 7S-150 conjugates showed a flat pattern, which showed that natural 7S and 7S-150 were in the amorphous state [9] whereas many sharp peaks were found in the XRD spectrum of free curcumin including 12.16°, 14.52°, 17.16°, 19.32°, 21.16°, 23.24°, 24.64°, and 26.08° in the 2θ range, suggesting that free curcumin was highly crystalline, which was consistent with the description in previous literature [35]. Interestingly, these sharp peaks disappeared in the XRD spectra of 7S-Cur and 7S-150-Cur, indicating that nanoencapsulation of 7S-Cur and 7S-150-Cur could inhibit the crystallization of curcumin, resulting in curcumin being an amorphous form within the nanocomplexes [35]. Zhang et al. [36] and Li et al. [21] also reported that after complexation with the pea protein or soy protein isolate, the characteristic XRD peaks of curcumin were absent in the nanocomplexes. Furthermore, due to the pH-shifting process, this resulted in the formation of NaCl crystals, and there were two new peaks (31° and 45°) that occurred in the spectra of the 7S-Cur and 7S-150-Cur nanocomplexes [37].

#### 3.3.5. Micromorphology and Visual Appearance of 7S-Cur and 7S-DEX-Cur Nanocomplexes

**Figure 5:**
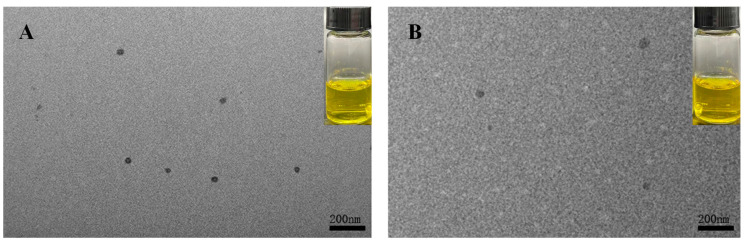
Transmission electron microscopy images of 7S-Cur (**A**) and 7S-150-Cur (**B**). The tube in each image shows the visual appearance of the corresponding nanocomplexes. 7S: soy β-conglycinin; 150: dextran (150 kDa); Cur: curcumin.

The TEM image and visual appearance of the two particles are presented in Figure 5. The particle size of the 7S-Cur nanocomplexes was around 60 nm smaller than of 7S-150-Cur (approximately 90 nm), which is in keeping with the results of DLS. Both the 7S-Cur and 7S-150-Cur nanocomplexes exhibited a spheroid shape with uniform dispersion, suggesting that the obtained nanocomplexes had a stable structure [7]. Meanwhile, the solutions of 7S-Cur and 7S-150-Cur were clear and transparent, suggesting that the curcumin nanocomplexes were uniformly dispersed in water. Furthermore, the 7S-150-Cur solution exhibited more distinct colors than the 7S-Cur solution, indicating that 7S-150 conjugates have higher LA for curcumin than nature 7S, consonant with the findings in Section 3.3.2.

#### 3.3.6. Thermal Stability of 7S-Cur and 7S-150-Cur Nanocomplexes

**Figure 6:**
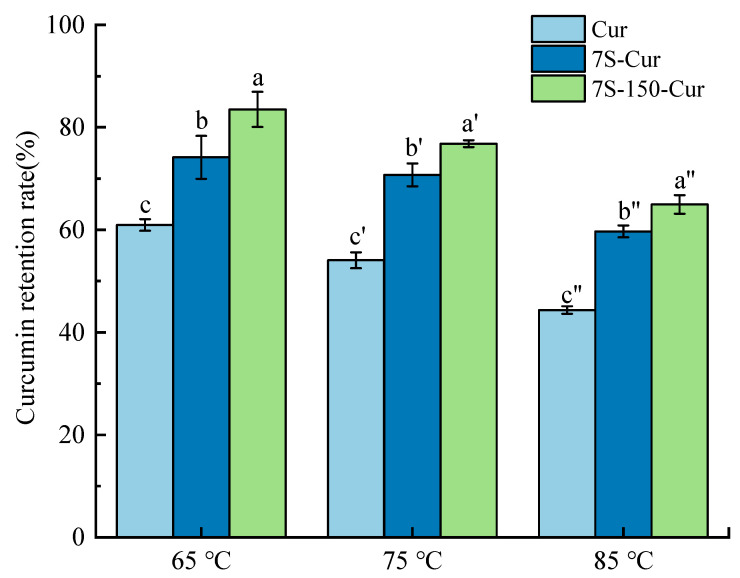
Heat stability of the free curcumin, 7S-Cur, and 7S-150-Cur nanocomplexes (different superscript (a–c), (a′–c′), and (a″–c″) letters in the figure indicate the significant differences between samples at the same heating temperature (*p* < 0.05), respectively). 7S: soy β-conglycinin; 150: dextran (150 kDa); Cur: curcumin.

Past studies have widely proven that curcumin has a poor thermal stability in aqueous solutions, which has severely limited the applications and bioavailability [23]. The curcumin retention rates after heating at 65 °C, 75 °C, and 85 °C for 30 min of the free and encapsulated curcumin were measured. As shown in Figure 6, under the same heating treatment, encapsulated curcumin exhibited a higher retention rate than free curcumin. For example, after heating at 65 °C for 30 min, the curcumin retention rate of 7S-Cur and 7S-150-Cur nanocomplexes were 74.14% and 83.50%, respectively, while that of free curcumin was only 60.95%. This phenomenon indicated that encapsulation within 7S/7S-150 could protect the chemically active groups of curcumin from thermal degradation to enhance the thermal stability of curcumin. Similar phenomena have also been described in other reports, for example, that complexation with soy protein or pea protein could alleviate curcumin thermal decomposition [36,38]. Meanwhile, compared with 7S-Cur, 7S-150-Cur exhibited a higher curcumin retention rate after the same heating treatment, which could be attributed to the addition of dextran. Polysaccharides have an excellent heating resistance, and Chen et al. [39] found that coating a chitosan layer on the surface of zein-curcumin nanocomposites could further improve the thermal stability of curcumin within the nanocomplexes.

#### 3.3.7. Storage Stability of 7S-Cur and 7S-150-Cur Nanocomplexes

**Figure 7:**
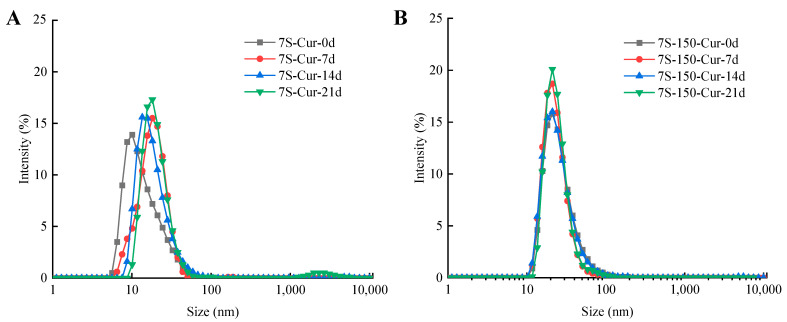
Changes in the particle size of the 7S-Cur (**A**) and 7S-150-Cur (**B**) nanocomplexes during the storage time (0–21 days). 7S: soy β-conglycinin; 150: dextran (150 kDa); Cur: curcumin.

As a potential delivery system for curcumin, the nanocomplexes should have a considerable stability throughout their shelf life. During storage, the particle size of 7S-Cur and 7S-150-Cur was monitored to determine whether the nanocomplexes had good long-term stability. As illustrated in Figure 7A, the particle size of 7S-Cur was gradually increased. In particular, at 21 days, large sized particles occurred (>1000 nm), indicating that 7S-Cur would aggregate during its long storage time. However, the particle size distribution (Figure 7B) of the 7S-150-Cur nanocomplexes did not vary much over the 0–21 day range, suggesting that the 7S-150-Cur nanoparticles had an excellent storage stability. This result could be interpreted as the fact that the attachment of dextran supplied sufficient steric hindrance to inhibit nanoparticle flocculation, thereby stabilizing the nanocomplexes during the long storage time. Fu et al. [40] also reported that bovine serum albumin-glucose-resveratrol nanoparticles showed better storage stability than bovine serum albumin-resveratrol nanoparticles.

## 4. Conclusions

In conclusion, the prepared 7S-150-Cur nanocomplexes effectively improved the water dispersion and stability of curcumin. The results of fluorescence quenching analysis and FITR illustrated that curcumin and 7S/7S-150 formed a nanocomplex spontaneously through non-covalent interaction. Glycation with dextran enhanced the binding affinity of 7S for curcumin, and 7S-150 exhibited higher LA and EE% of curcumin than natural 7S. The XRD spectrum revealed that the encapsulated curcumin within 7S/7S-150 was in an amorphous state. 7S-150-Cur nanocomplexes showed better thermal stability and storage stability than the 7S-Cur nanocomplexes. Overall, this study presented preliminary information on the use of glycated proteins as nanocarriers for hydrophobic nutrients to improve their physicochemical stability, and provided a reference for the future applications of hydrophobic nutrients in the food industry.

## Figures and Tables

**Table 1:** Degree of glycation (DG%) of 7S and 7S-DEX conjugates (7S-40, 7S-70, 7S-150, 7S-500).

Samples	DG (%)
7S	-
7S-40	16.19 ^a^
7S-70	12.31 ^b^
7S-150	11.13 ^c^
7S-500	6.79 ^d^

Different superscript letters in the same column indicate significant differences (*p* < 0.05). 7S: soy β-conglycinin; DEX: dextran; 40: dextran (40 kDa); 70: dextran (70 kDa); 150: dextran (150 kDa); 500: dextran (500 kDa).

**Table 2:** The Z-average diameter, polydispersity index (PDI), ζ-potential, encapsulation efficiency (EE%), and loading amount (LA) of the 7S-Cur and 7S-150-Cur nanocomplexes prepared at 25 °C.

Samples	Z-Average Diameter (nm)	PDI	ζ-Potential (mV)	EE% (wt%)	LA (μg/mg)
7S-Cur	75.11 ± 3.50 ^b^	0.24 ± 0.0124 ^b^	−17.77 ± 1.14 ^a^	51.15 ± 1.07 ^b^	12.41 ± 0.26 ^b^
7S-150-Cur	84.37 ± 1.30 ^a^	0.27 ± 0.0925 ^a^	−7.88 ± 0.43 ^b^	87.51 ± 3.81 ^a^	16.06 ± 0.70 ^a^

Different superscript letters in the same column indicate significant differences (*p* < 0.05). 7S: soy β-conglycinin; 150: dextran (150 kDa); Cur: curcumin.

## Data Availability

Data are included within the study.
